# An intriguing approach toward antibacterial activity of green synthesized Rutin-templated mesoporous silica nanoparticles decorated with nanosilver

**DOI:** 10.1038/s41598-023-33095-1

**Published:** 2023-04-12

**Authors:** Milad Abbasi, Razieh Gholizadeh, Seyed Reza Kasaee, Ahmad Vaez, Shreeshivadasan Chelliapan, Fouad Fadhil Al-Qaim, Issa Farhan Deyab, Mostafa Shafiee, Zahra Zareshahrabadi, Ali Mohammad Amani, Sareh Mosleh-Shirazi, Hesam Kamyab

**Affiliations:** 1 Department of Medical Nanotechnology, School of Advanced Medical Sciences and Technologies, Shiraz University of Medical Sciences, Shiraz, Iran; 2 Department of Materials Science and Engineering, Shiraz University of Technology, Shiraz, Iran; 3 Shiraz Endocrinology and Metabolism Research Center, Shiraz University of Medical Sciences, Shiraz, Iran; 4 Department of Tissue Engineering and Applied Cell Sciences, School of Advanced Medical Sciences and Technologies, Shiraz University of Medical Sciences, Shiraz, Iran; 5 Engineering Department, Razak Faculty of Technology and Informatics, Universiti Teknologi Malaysia, Jln Sultan Yahya Petra, 54100 Kuala Lumpur, Malaysia; 6 College of Science for Women, University of Babylon, Hilla, Iraq; 7 Medical Physics Department, Al-Mustaqbal University College, Hillah, Babil 51001 Iraq; 8 Basic Sciences in Infectious Diseases Research Center, Shiraz University of Medical Sciences, Shiraz, Iran; 9 Department of Biomaterials, Saveetha Dental College and Hospital, Saveetha Institute of Medical and Technical Sciences, Chennai, 600 077 India; 10 Malaysia-Japan International Institute of Technology (MJIIT), Universiti Teknologi Malaysia, Jalan Sultan Yahya Petra, 54100 Kuala Lumpur, Malaysia

**Keywords:** Biotechnology, Materials science, Nanoscience and technology

## Abstract

In recent years, mesoporous silica nanoparticles (MSNs) have been applied in various biomedicine fields like bioimaging, drug delivery, and antibacterial alternatives. MSNs could be manufactured through green synthetic methods as environmentally friendly and sustainable synthesis approaches, to improve physiochemical characteristics for biomedical applications. In the present research, we used Rutin (Ru) extract, a biocompatible flavonoid, as the reducing agent and nonsurfactant template for the green synthesis of Ag-decorated MSNs. Transmission electron microscopy (TEM), zeta-potential, x-ray powder diffraction (XRD), fourier transform infrared (FTIR) spectroscopy analysis, scanning electron microscopy (SEM), brunauer–emmett–teller (BET) analysis, and energy-dispersive system (EDS) spectroscopy were used to evaluate the Ag-decorated MSNs physical characteristics. The antimicrobial properties were evaluated against *Staphylococcus aureus (S*. *aureus), Escherichia coli* (*E*. *coli),* and also different types of candida. The cytotoxicity test was performed by using the MTT assay. Based on the findings, the significant antimicrobial efficacy of Ru-Ag-decorated MSNs against both gram positive and gram negative bacteria and different types of fungi was detected as well as acceptable safety and low cytotoxicity even at lower concentrations. Our results have given a straightforward and cost-effective method for fabricating biodegradable Ag-decorated MSNs. The applications of these MSNs in the domains of biomedicine appear to be promising.

## Introduction

Many natural compounds, including alkaloids, terpenoids, organic acids, polysaccharides, flavonoids, anthraquinones, saponins, volatile oils, and others, have demonstrated promising antiviral and antibacterial effects in recent years^1^. A class of key natural organic chemicals and secondary metabolites are flavonoids, which have diverse pharmacological effects, biological activities, as well as antibacterial capabilities^2^.

3, 3′, 4′, 5, 7-pentahydroxyflavone-3-rhamnoglucoside (Rutin) is the flavonol that can be found abundantly in plants, for instance tea, apple, buckwheat, and passion flower. It is an extremely important nutritional ingredient in food products^3^. Rutin (Ru), also known as sophorin, quercetin-3-rutinoside, and rutoside, is the flavonoid glycoside found in buckwheat that is derived from citrus fruits^4^. This flavonol gets its name from the plant Ruta graveolens that contains Ru as well. Ru is a glycoside composed of the disaccharide rutinose flavonolic and the aglycone quercetin, according to its chemical makeup^5,6^. The compound has been revealed to have several pharmacological properties, containing vasoprotective, anticarcinogenic, antioxidant, neuroprotective, cardioprotective, cytoprotective, and antibacterial effects^7,8^.

Nanomaterials have various applications in biology, medicine, electrical engineering, and chemical research. The size and shape of nanostructures are the most important criteria in determining their overall effectiveness^9–12^. Silver (Ag) nanoparticles have at least one dimension ranging between 1 and 100 nm in size. Given their extraordinary capabilities, Ag nanoparticles (NPs) have attracted a significant deal of research in the fields of biomedical applications, catalysis, and chemistry. Ag nanoparticles are a very adaptable nanomaterial featuring antibacterial characteristics that can be used in many applications^13,14^. Furthermore, they have been revealed to have antifungal and anti-inflammatory properties. The Ag nanoparticles’ antibacterial properties are related to their ability to attach to cell membranes and then their release into bacterial cells, both of that contribute to their high level of activity. Due to their increased surface energy, Ag nanoparticles, on the other hand, are extremely quickly oxidized and aggregated, limiting their antibacterial efficacy in clinical settings^15,16^. To address these challenges, several Ag-carried substances, such as zeolite, carbon, silica, and titanium dioxide, have been created to enhance effectual as well as multipurpose functions in the areas of infection prevention and control, tissue regeneration, wound healing, and the treatment of oral disease^16–20^.

Mesoporous silica nanoparticles (MSNs) have been widely used as suitable substances for carrying nanosilver because of their simplicity of surface modification, excellent biocompatibility, large surface area, and uniform porous framework^21^. MSNs can have a controlled release of Ag ions and safeguard nanosilver from agglomeration, leading to prolonged efficiency while significantly lowering undesirable toxic effects. Ag nanoparticles positioned within pore spaces as well as onto the surfaces of MSNs have demonstrated enhanced antibacterial effectiveness^22,23^. This is owing to the deceased dimensions of the Ag nanoparticles and also the quicker liberation of Ag ions in comparison with those covered with mesoporous silica shells to create core–shell architectures. The uncontrollable and slower biodegradation characteristics of traditional MSNs make it difficult to employ them in clinical settings in the future. Not only did Ag-coated mesoporous organosilica nanomaterials demonstrate good activity against an extensive variety of bacterial films and bacteria, but they also demonstrated glutathione-responsive disintegration, which allowed for the release of Ag ions^24,25^. The decorating of Ag nanoparticles on MSNs, which is a distinct synthesis phase including the production of MSNs, the Ag nanomaterial reduction, and template removal, is particularly remarkable since it can take up to 2–3 days to complete in most situations^26^.

Despite the fact that there are several techniques to synthesize nanostructures^16,27–30^, their environmentally friendly green synthesis has gained attention over recent years^27,31^. When it comes to reductants, green synthesis or novel synthesis methods can make use of a wide range of resources such as plant extracts, microorganisms, biomass, and many other types of chemicals^32,33^. There have been reports of the use of biomass, particularly flavonoids, as a technique for the reduction of Ag^+^ ion to silver nanoparticles^34,35^. Flavonoids have significant application potential due to the large variety of sources, as well as excellent synthesis effectiveness.

In the present study, Ru is used to reduce Ag ions into nanoparticles, which is a green synthesis technique for the fabrication of Ru-based Ag-decorated MSNs. Ru not only helps make MSNs by acting as a nonsurfactant template, but it also helps make homogeneous Ag nanoparticles by acting as a reduction agent. This process does not involve the use of any hazardous chemicals. Compared to the traditional approaches, this procedure is more cost-effective, simpler to run, and more ecologically friendly, therefore considered “green”. The antimicrobial effects of Ru-templated Ag-decorated MSNs have been investigated in this study. This investigation will make a significant contribution to natural biomass exploration as well as its application in nanotechnology and nanoscience.

## Materials and method

### Materials

Rutin (Ru), tetraethoxysilane (TEOS), brain heart infusion agar (BHI), silver nitrate, ammonium hydroxide, sabouraud dextrose agar (SDA), and ethanol were purchased from Merck Company. Furthermore, 3-(N-morpholino) propane sulfonic acid (MOPS) and RPMI-1640 media were purchased from Sigma Company.

### Synthesis of biocompatible Ru-templated Ag-decorated MSNs

#### Synthesis of MSNs

Rutin (Ru) is a flavonoid that could be found in most citrus fruits and has been defined to have significant anticancer, anti-inflammatory, and antioxidant properties^36,37^. The green synthesis method was used for the production of the nanoparticles (NPs) by applying Ru (C_27_H_30_O_16_) (Fig. S1) extract as a reducing agent that was being adjusted to reserve the advantage in excess of chemical approaches. In the green synthesis method, no chemicals were performed and even the Ru’s properties were transferred to the NPs^32^.

First, 200 ml of ethanol was poured into a beaker and placed on a stirrer. Then 100 ml of ammonia was added to the solution, and then 6.4 ml of Ru extract (0.1 mM) was added to it. In the next step, 1.2 ml of TEOS was added to the solution and then stirred for 2 h that the color change from white to yellow.

After 2 hours, the prepared solution was poured into a falcon and centrifuged at 10^4^ rpm at 7 °C for 10 min. At the end of the process, the supernatant of the prepared solution was removed, and the falcon was placed inside the oven at 30 °C. The dried MSNs were then used for later applications. We had four falcons containing MSNs, which we divided into four, meaning that the volume of each falcon containing MSNs was 0.07 g.

#### Preparation of Ag-decorated Ru-templated MSNs

First, 0.07 g of the MSNs were added to 14.88, 14.80, 14.72, and 14.59 ml of deionized water to produce 3, 5, 7, and 10% Ag-decorated MSNs, respectively, and subsequently sonicated at 70 to 80 mV for 3 min to disperse the solution. The resulting solution was then stirred, and 0.12 ml of Ag (0.1 M) was added to the solution and placed at the temperature of 40 °C to 50 °C for 5 h. In the next step, the prepared solution was placed in the oven at the temperature of 30 °C for 24 h. The obtained powder was then used for further tests.

### Characterization methods

The transmission electron microscope (TEM, Philips CM 10) was employed to confirm the formation, morphology, and visual appearance analysis of Ru-templated Ag-decorated MSNs. TEM samples were provided with well-scattered NPs drops on the 300-mesh carbon-coated copper grid^38^. Dynamic light scattering method (DLS, MALVERN Zen3600) was employed to determine the size distribution of NPs. The scanning electron microscope (SEM, Philips XL30) which was equipped with Energy-dispersive X-ray spectroscopy (EDS) were employed to investigate the morphology and structure of NPs. For the purpose of analyzing the structure of the nanoparticles, a PANalytical X'Pert Pro X-ray diffractometer was employed. The Fourier transform infrared spectroscopy (FTIR) analyses were used by a (Perkin-Elmer spectrometer), at 4000–400 cm^−1^. The absorption peak of Ru-templated Ag-decorated MSNs was obtained by a UV–Vis spectrophotometer (Varian, model; Carry 100) at 300–700 nm.

### Antimicrobial tests

The antimicrobial effects of the Ag-MSN NPs against 8 CentraalBureau voor Schimmel cultures (CBS) and American Type Culture Collection (ATCC) and strains of fungi and bacteria, including *Candida glabrata* (*C. glabrata,* ATCC 90,030), *Candida albicans* (*C. albicans,* CBS562), *Candida tropicalis* (*C. tropicalis,* ATCC 750), *Candida parapsilosis* (*C. parapsilosis,* ATCC 4344), *Candida dubliniensis* (*C. dubliniensis,* CBS 8501), *Candida krusei* (*C. krusei,* ATCC 6258), *Escherichia coli* (*E. coli),* and *Staphylococcus aureus (S. aureus)* and were used in this study^38^.

Yeasts were sub-cultured on SDA and then incubated at 32 °C for 24 h. Bacteria were grown on BHI and incubated at 37 °C for 24 h. Furthermore, the Minimum inhibitory concentrations (MICs) values of the Ag-MSN NPs against the evaluated fungi and bacteria were defined by using the broth microdilution technique according to the guidelines of Clinical and Laboratory Standards Institute (CLSI) documents. To evaluate the antimicrobial activities, serial dilutions of the Ag-MSN NPs and Ag NPs (0.5–256 µg/ml) were prepared in 96-well microtiter plates by MOPS with RPMI-1640 media (buffered at pH 7.0. The bacteria and fungi stock inoculum suspensions were prepared by suspending their colonies in sterile 0.85% NaCl (5 ml). Furthermore, the suspensions’ turbidity was adjusted to 0.5 MacFarland standards at the wavelength of 530 nm to yield a stock suspension of 1–5 × 10^6^ cell/ml by the spectrophotometric method. The bacteria and yeasts’ working suspension was prepared by using a 1/100 and 1/1000 dilution of their stock suspension with suitable broth media, respectively. Subsequently, the addition of 0.1 ml of the working inoculums to each well, the plates were also incubated for 24–48 h at 32 and 37 °C in the humid atmosphere for the bacteria and yeasts, respectively. The first column’s well of the microtiter plate (with 200 µl of the uninoculated medium) were attribute as a sterility control (blank) and growth controls having medium with inoculums without the developed nano-sized drug were used. Furthermore, the lowest concentration of each component that inhibits the visible growth of microorganisms was defined as MIC.

Additionally, for the investigation of the minimum fungicidal concentration (MFC) and minimum bactericidal concentration (MBC), 10 µl of the media from the wells revealing no visible growth was cultured on BHI for bacteria and SDA for fungi. The tube containing the medium with microorganisms but without any sample and the tubes containing the medium without any microorganisms were considered as the positive and negative controls, respectively. The MFCs and MBCs were revealed as the lowest concentration of studied antimicrobial compounds, indicating fewer than 4 colonies or no growth that relates to the microorganisms’ mortality of 98% in the initial inoculums. Each experiment was done in triplicate.

### Cytotoxicity and cell culture endpoints

The cytotoxicity test was performed by using the MTT assay that is related to the activity of mitochondrial dehydrogenase^39^. The cytotoxicity evaluation was performed by using fibroblast cells achieved from the Pasteur Institute’s National Cell Bank of Iran. Furthermore, the cell line was cultured RPMI-1640 medium (Gibco, Scotland) containing 1% penicillin/streptomycin and 10% fetal bovine serum (FBS, PAA, Austria) in the flask of 5% CO_2_ at 37 °C.

After that, the cell line culture was washed with phosphate-buffered saline (PBS) and separated from the flask by contacting with trypsin/EDTA for a short time. Furthermore, the cell’s density was determined by scattering trypan blue dye on extracted cell slides.

100 µl of complete culture media having around 1 × 10^5^ cells were put in each well of the 96-well sterile dish, incubated for 24 h, and subsequently treated with different concentrations of Ru-Ag-MSN (6.25, 12.5, 25, 50, 100, 150, 200, 250, 300, and 400 μg/ml) incubated for 24, 48, and 72 h. Furthermore, the control was determined culture tube that did not add any Ru-Ag-MSN. Whenever the preferred duration was obtained, the medium was then withdrawn, and 100 ml of the MTT material (10% of each well’s total volume) was added in the same manner.

Furthermore, DMSO (Merck, Germany) (100 ml) was added to each well, then the mixture was gradually pipetted several times with a sampler to completely dissolve the formazan crystals in the DMSO solution. The test absorbance at 650 and 570 nm was determined by a plate reader. The cell viability percentages of each sample in different concentrations were evaluated by the following equation^32^:

$$Cell \,\, viability \,\, \left(\% \right)= \frac{AbSt570-AbSt650}{AbCon570-AbCon650}\times 100.$$

The samples’ light absorbance is depicted by AbCon650 and AbCon570 (at 650 and 570 nm, respectively).

### Statistical analysis

Data were investigated by the SPSS software version (20.0, developed by SPSS Inc. in Chicago, Illinois, USA) in a p-value of less than 0.05 was revealed statistically significant. The most prevelant statistical techniques and tools, like the mean, standard deviation, and ontest ANOVA, were applied for data analysis.

## Results and discussion

**Figure 1:**
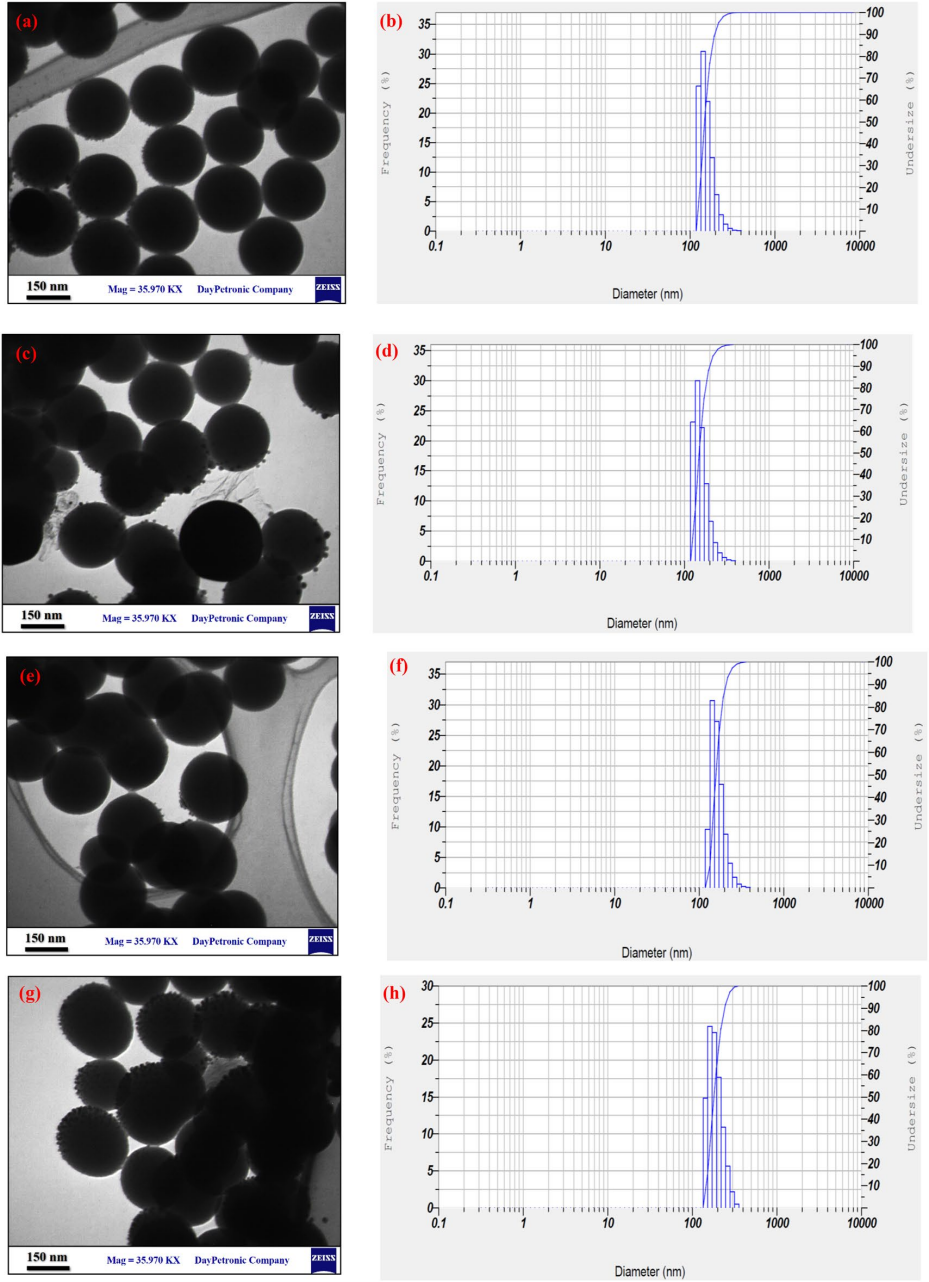
TEM micrograph and DLS results of (**a**,**b**) 3, (**c**,**d**) 5, (**e**,**f**) 7, and (**g**,**h**) 10% Ag-decorated MSNs.

It was possible to investigate the morphology, shape, and size of the Ru-templated Ag-decorated MSNs by using TEM. As illustrated in Fig. 1, TEM images of MSNs synthesized under optimal circumstances were obtained, and the synthesized MSNs were revealed to be predominantly spherical in shape. Furthermore, the Ru-templated Ag-decorated MSNs were synthesized in a single step using a one-step approach. When compared to earlier approaches, our method was more environmentally friendly and advantageous since it did not necessitate the preceding manufacture of Ag nanoparticles (NPs) or the removal of MSN templates. This study used Ru as a non-surfactant template that was integrated into the silica structures by the use of condensing TEOS. It was found that the Ru-templated MSNs formed with just the template removal had a homogenous spherical shape featuring an average diameter of around 150 nm. Since the pores of MSNs are decorated with Ag NPs, the pores couldn’t be observed in the TEM analysis (Fig. S2). Ru’s supramolecular composition could can well have served as a scaffold for the polymerization of silica precursors surrounding it, resulting in the pores’ formation through electrostatic interactions and hydrogen bonds. Also, Ru was utilized as a reduction and stabilizing agent during the green synthesis of Ag NPs; after the addition of silver nitrate, the reduction procedure took place in situ. TEM images demonstrated that Ag NPs with the average diameter of 2–10 nm were decorated on the MSNs surfaces successfully (Fig. S3). Furthermore, as the percentage of Ag NPs increases, the Ag-decorated Ru-templated MSNs’ size increases (Fig. 1a, c, e and g).

Furthermore, Fig. 1 represents the particle size distribution diagram for Ag-decorated Ru-templated MSNs obtained from the DLS method, representing that as the percentage of Ag NPs increases from 3 to 10%, the mean size of NPs increases from 155 to 188 nm, respectively. These results have a good agreement with the TEM results. The polyphenols’ existence in the plant, which acts as a covering agent inhibits the particles’ aggregation that is the green synthesis technique’s significant advantage in comparison with the chemical synthesis route^24,25^.

**Figure 2:**
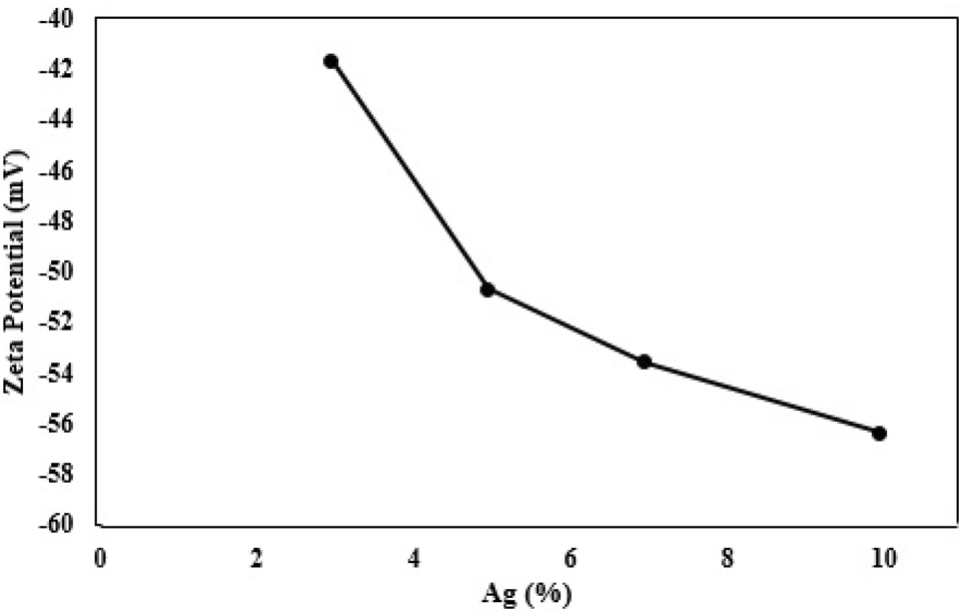
Zeta potential value of Ag-decorated Ru-templated MSNs.

The zeta potential is the index of surface charge potential and a important parameter to define the stability of nanoparticles in the suspension^40^. The zeta potential values of Ag-decorated Ru-templated MSNs are displayed in Fig. 2. The zeta potential’s negative value of the Ag NPs reveals their significant and long-term stability in the suspension. Moreover, the Ag NPs had coated with anionic compounds, and consequently, the repulsive force of interparticle electrostatic led to the preclusion of the aggregation of the nanoparticles. Previous studies have indicated that the NPs are attached to the variety of biomolecules and functional groups such as phenols, proteins, and carbohydrates that assistance to stabilize and reduce metal ions into NPs^27,41,42^. The presence of carbonyl functional group (Fig. 6) makes the surface of Ag NPs negative charge. The higher zeta potential’s negative value indicates more stability and interparticle repulsion.

**Figure 3:**
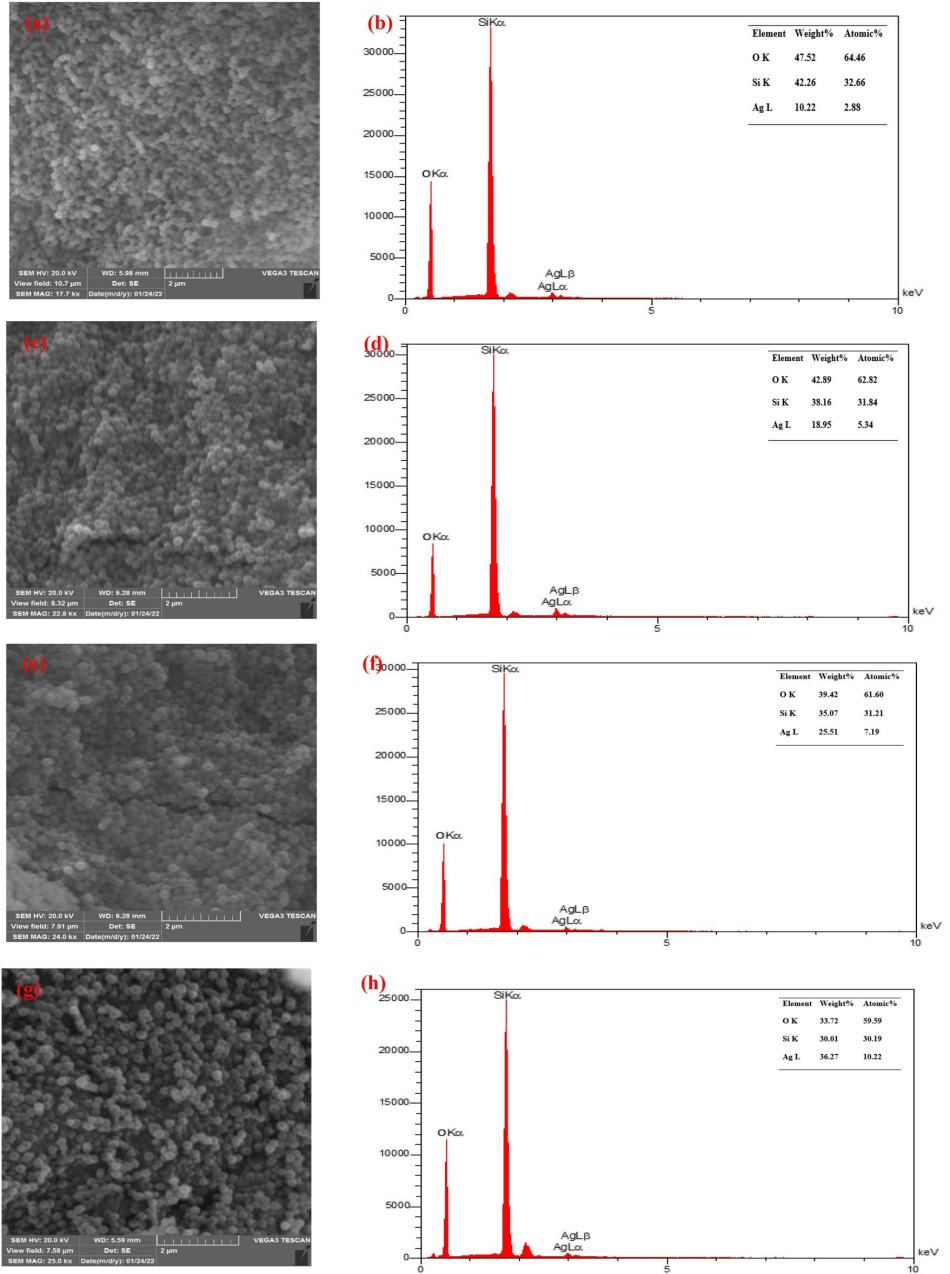
SEM micrograph and EDS patterns of (**a**,**b**) 3, (**c**,**d**) 5, (**e**,**f**) 7 and (**g**,**h**) 10% Ag-decorated MSNs.

The size and morphology of the MSNs were evaluated using SEM (Fig. 3), which revealed that Ag-decorated Ru-templated MSNs did not agglomerate and were separated by reason of the existence of Ruthat acts as the covering agent. Furthermore, as the percentage of Ag NPs increments, the Ag-decorated Ru-templated MSNs’ size increments, which confirms the TEM and DLS results.

The elemental structure of the Ag-decorated Ru-templated MSNs is described in Fig. 3. Silver, oxygen, and silicon are all determined in the spectra, as revealed by the MSNs and Ag structures’ elemental composition. No impurities or contamination were detected.

**Figure 4:**
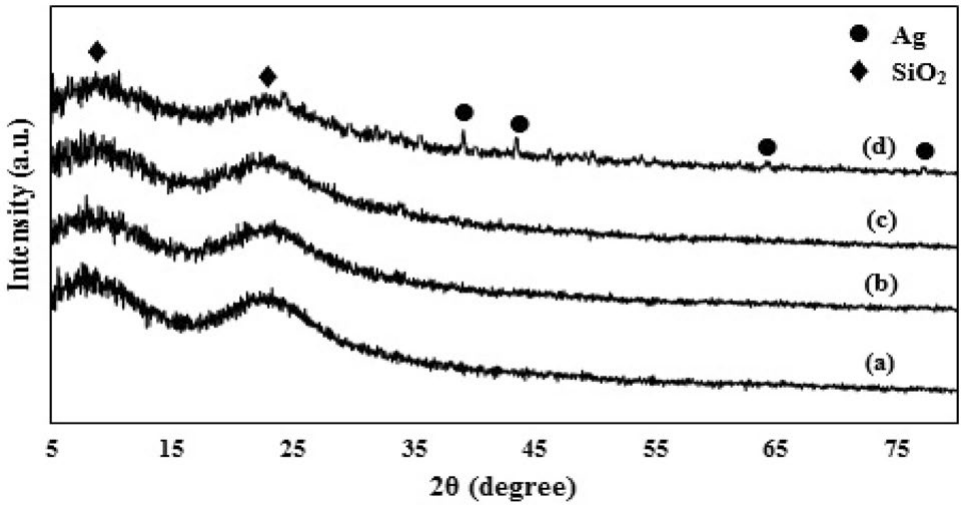
XRD pattern of (**a**) 3, (**b**) 5, (**c**) 7 and (**d**) 10% Ag-decorated MSNs.

The X-ray diffraction patterns of Ag-decorated Ru-templated MSNs are observed in Fig. 4. Two broad peaks revealed at 9° and 23° confirm the amorphous structure of MSNs^43,44^. Furthermore, the x-ray diffraction peaks at two theta are 39.1°, 43.5°, 64.4°, and 77.3°, which are reflected from the silver crystal planes (111), (200), (220), and (311), respectively, that represent the FCC (face-centered cubic) structure of Ag NPs. Furthermore, by increasing the percentage of silver up to 10%, the XRD peaks of silver could be clearly detected. At lower percentages of silver nanoparticles, the corresponding XRD peaks are weaker (Fig. S4).

Based on the BET analysis, surface parameters such as the surface area, mean pore diameter, and total pore volume of different Ag-decorated Ru-templated MSNs were revealed. As shown in Table 1, the results proved the successful preparation of porous mesoporous materials.

**Table 1:** Surface parameters of Ag-decorated Ru-templated MSNs.

Ag (%)	S_BET_ (m^2^g^−1^)	V_P_ (cm^3^g^−1^)	D_P_ (nm)
3	239.99	0.308	5.13
5	246.64	0.293	4.75
7	267.85	0.312	4.66
10	396.16	0.333	3.36

**Figure 5:**
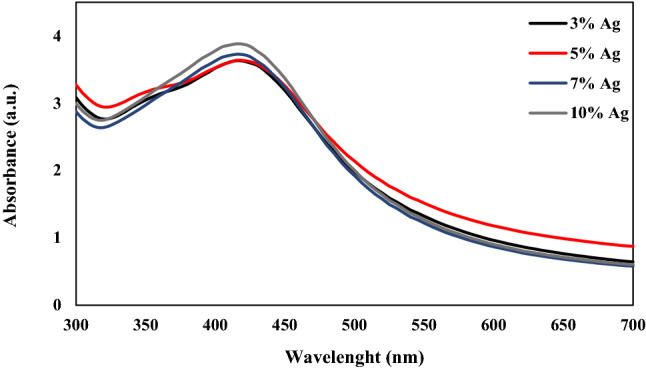
UV–vis spectroscopy of Ag-decorated Ru-templated MSNs.

The UV–vis absorption spectra of the Ag-decorated Ru-templated MSNs are displayed in Fig. 5. The surface plasmon resonance (SPR) absorbance is significantly sensitive to the nature, size, and shape of the surrounding medium and particles^38^. There was a distinct SPR peak at around 420 nm in the UV–Vis absorption spectrum of the Ag-decorated Ru-templated MSNs, corresponding to the formation of Ag NPs^28,38,42^.

**Figure 6:**
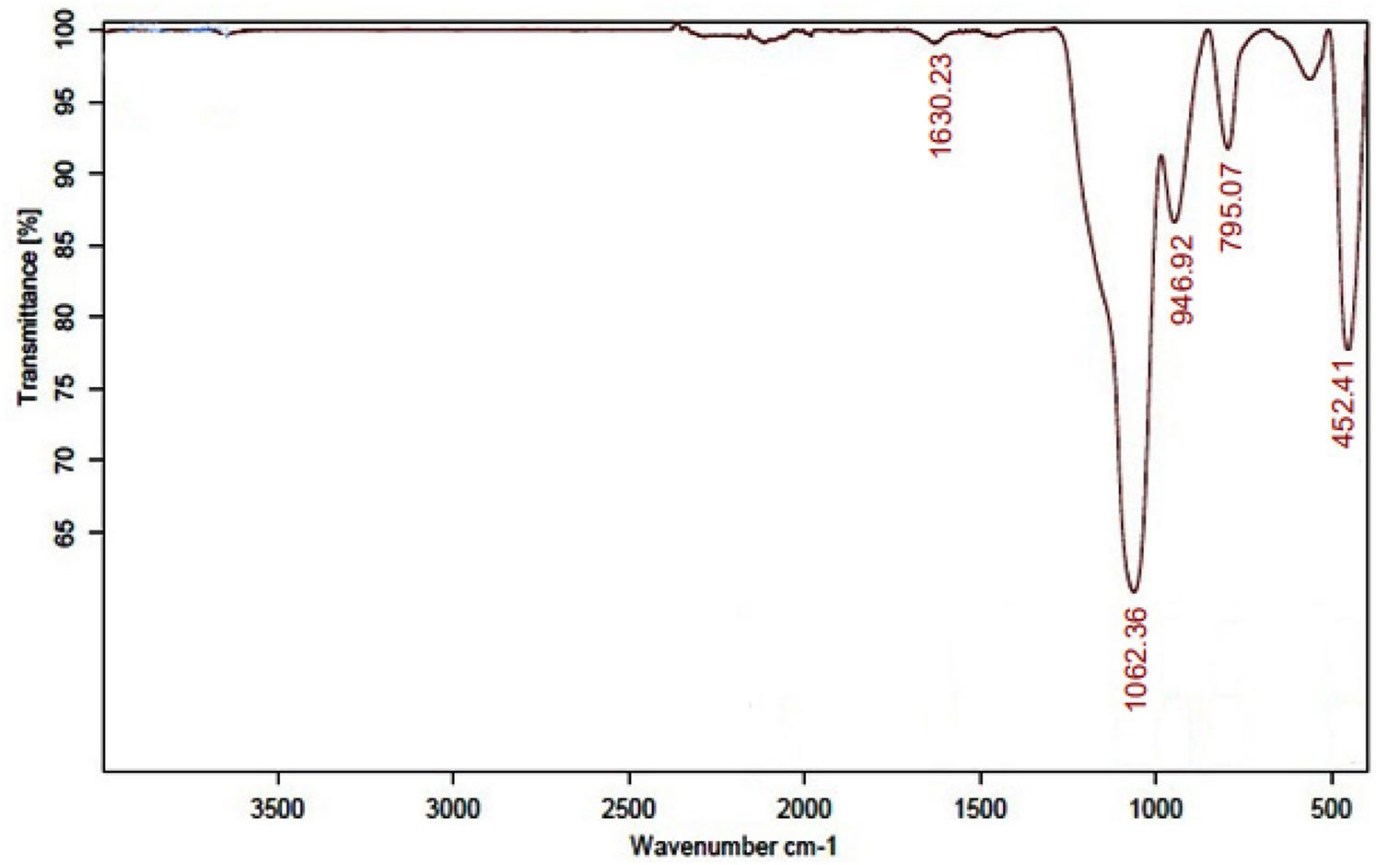
FTIR spectrum of Ag-decorated Ru-templated MSNs.

In order to determine the functional groups present surrounding the synthesized Ag-decorated Ru-templated MSNs, FTIR spectroscopy was performed in the wavelength region between 4000 and 400 cm^−1^. As displayed in Fig. 6, the characteristic FTIR spectrum of nanomaterials exhibits three distinctive bands of silica in the range of 400 to 1200 cm^−1^. The bending vibrations of Si–O–Si are responsible for the sharp band at 452.41 cm^−1^, while the symmetric vibrations of Si–O–Si are responsible for the band at 795.07 cm^−1^, and a broad band at 1062.36 cm^−1^ is responsible for the asymmetric vibrations of Si–O–Si of silica. Furthermore, the peak at 946.92 cm^−1^ in the spectrum could be related to the bending vibration absorption of Si−OH. The carbonyl stretching of the proteins is thought to be responsible for the peak that emerged at 1630.23 cm^−1^. The existence of this functional group increases the stability and its biological efficacy^27^.

The antimicrobial results confirmed that different concentrations of the Ag-decorated Ru-templated MSNs reveal excellent and significantly higher antifungal and antibacterial properties against both gram negative and gram positive bacteria and diverse types of candida in compare with traditional silver nanoparticles, even in lower concentrations (Table 2). Previous studies have detected that pure Ag NPs exhibit antibacterial and antifungal characteristics at higher concentrations (> 128 mg/ml) in comparison to Ag-MSNs^38,45,46^. However, at this concentration, Ag NPs could be toxic for normal cells due to cell penetration and destruction of normal cell structures.

**Table 2:** Antimicrobial function of different Ag concentrations of green synthesized Ru-Ag-MSN (Minimum inhibitory concentration (MIC), Minimum bacteriocidal or fungicidal concentration (MMC).

Fungi & bacteria	Organisms	ATCC	3% Ag		5% Ag		7% Ag		10% Ag	
			MIC 90 (μg/ml)	MMC (μg/ml)	MIC 90 (μg/ml)	MMC (μg/ml)	MIC 90 (μg/ml)	MMC (μg/ml)	MIC 90 (μg/ml)	MMC (μg/ml)
Standard strains	*C*. *albicans*	(CBS562)	100	100	50	100	50	100	25	50
	*C*. *tropicalis*	(ATCC750)	150	200	150	200	100	150	50	100
	*C*. *krusei*	(ATCC 6258)	150	250	150	200	100	200	100	150
	*C*. *glabrata*	(ATCC 90,030)	50	100	50	100	25	50	25	50
	*C*. *parapsilosis*	(ATCC 4344)	150	300	150	200	100	200	100	150
	*C*. *dubliniensis*	(CBS 8501)	100	150	100	150	50	100	25	50
	*E*. *coli*	A (11,229)	50	100	25	100	12.5	50	4.2	25
	*S*. *aureus*	A (25,923)	50	100	25	100	12.5	50	6.25	25

Some possible mechanisms could be responsible for the antimicrobial properties of Ag NPs. In fact, bacteria’s cell walls contain glycoproteins with negative charges that can simply attach to positive-charged nanoparticles (Ag NPs)^47^. The attachment of the Ag NPs to the microbial cell membrane can change the charge and permeability of membrane, which consequently destroys the cell wall. However, this mechanism is more prominent in the antifungal function of silver NPs.

In addition to cell membrane destruction, the cytoplasmic ROS generation play the major role in antibacterial properties of Ag NPs that can induce critical bacterial DNA damages resulting in bacterial death. Moreover, the interaction between Ag^+^ NPs and phosphate components in the bacterial cytoplasm could lead to the formation of stable complexes that disrupt critical bacterial enzymes^48^.

The superior and excellent antibacterial function of Ag-decorated Ru-templated MSNs against both gram positive and negative bacteria might be related to the active and steady release of Ag NPs from the Ru-templated MSN platform in contact with the bacterial membrane^45^.

Ag NPs could adhere to bacterial membranes and destruct cell walls. However, they may also aggregate on bacterial surfaces or in the media, which decreases their antimicrobial function over time^49^. Also, leakage of bacterial death components could aggregate the Ag NPs on remnant cell walls or alter the matrix environment that reduces the antimicrobial effect of Ag NPs over the time^50,51^.

Of course, the survived remaining bacteria begin to duplicate and cause the additional decrease in antimicrobial function of Ag NPs. Furthermore, the uniform distribution of Ag NPs with small sizes on the silicon substrate is a significant factor for improving the antimicrobial properties of Ag NPs and decreasing their cytotoxicity for normal cells^52^.

The supporting matrix (MSN) could improve the antimicrobial function of Ag NPs through protection of separated Ag NPs from aggregation, that each NP can effectively adhere to bacterial or fungal cell membrane. Ag decorated MSN can discharge Ag ions in a steady manner to increase antibacterial and antifungal effects without time dependent decrease efficacy^53^.

Consistent with previous studies, the antibacterial and antifungal functions of Ag nanoparticles followed a dose-dependent manner^38,45^. By increment the concentration of Ag NPs in the Ag-MSN composite, the MIC values against both types of bacteria were presented at lower concentrations.

By comparisons the results of other researches we detected that Ru-based Ag-decorated-MSNs reveal effective antimicrobial function in lower MIC and MBC values against certain types of bacteria and fungi in comparison with Ag-decorated MSNs^54–57^. It is suggested that adding Ru extract to silicon mesopore could enhance the antimicrobial characteristics of Ag-MSN.

The flavonoid compound in Rutin extract has been revealed to have several pharmacological properties, including anticarcinogenic, antioxidant, cardioprotective^7,8,38^.

Moreover, rutin extract has been reported to reveal antimicrobial function against both gram positive and negative bacteria either alone or in combination with other antibacterial agents such as aminopenicillanic acid^58,59^.

The phenolic component of the Rutin extract play an important role in its antimicrobial function and attenuation of the pathogenicity^60^. Different mechanisms were detected for antimicrobial function of the Rutin extract Flavonoids. The proposed mechanisms are as follows, alteration of the membrane permeability and disruption of cytoplasmic membrane function, prevention of bacterial DNA gyrase activity, restriction of nucleic acid synthesis and interfere with energy metabolism^58,61^.

Also, it has been detected that Ag NPs with smaller size could induce faster Ag ^+^ ion release from the matrix^42^. In our study, the Ag NP size on the MSN platform was around 2 − 10 nm that increased antimicrobial properties in comparison with larger Ag NPs. However, in most previous researches, the Ag NPs’ diameter on silica materials from 20 to 50 nm^38^.

According to Fig. 1, revealed efficient and steady implanted Ag NPs within the Ru-silica mesoporous platform (MSN) that could increase the antimicrobial function through the protection of Ag NPs from aggregation in addition to slow-releasing Ag ions from the component that promises to have more impressive application in the biomedical field.

It is recommended that Ru not only plays an assistance role in producing MSNs by acting as a nanosurfactant template, but it also helps make homogeneous Ag nanoparticles by acting as a reduction agent.

The antimicrobial results of the study indicated the nearly equivalent MIC of Ag-MSN for both gram positive *S*. *aureu*s and gram negative *E. coli* at 3, 5, and 7% Ag concentration (100, 50, and 25 μg/ml, respectively). However, at the concentration of 10% Ru-Ag-MSN, *E. coli* showed more susceptibility to Ag NPs than Staph areos (4.2 and 6.25 µg/ml, respectively).

It may be as a result of the additional negatively charged lipo-polysaccharide layer coating the *E*. *coli* cell membrane, that enhances Ag NPs cell attachment^62^. This point is more prominent at higher concentrations (10%) of Ag on Ag-decorated MSN, where the lower concentrations of Ag showed no significant difference between the MIC values of these two types of bacteria.

The results of antifungal tests showed that Ru-Ag MSN has effective antifungal characteristics against different types of candida (Table 2).

Previous research has found that Ag NPs can elevate an antifungal function by interrupting fungal cell membrane integrity, leading to the destruction of the membrane structure. Also, it can inhibit the normal budding process of fungi^63^. Omran et al. detected that Ag NPs can affect fungal nucleus through inhibition of some critical enzymes that play a role in DNA replication, such as DNAases, resulting in DNA damage and eventually cell death^64^.

The considerable antifungal properties of Ru-Ag MSN especially against some drug resistant types of candida as well as excellent antibacterial characteristics, could promising a new insight to produce Ag based nanomaterials with improved antimicrobial properties for biomedical applications such as medical devices or wound dressing agents.

**Figure 7:**
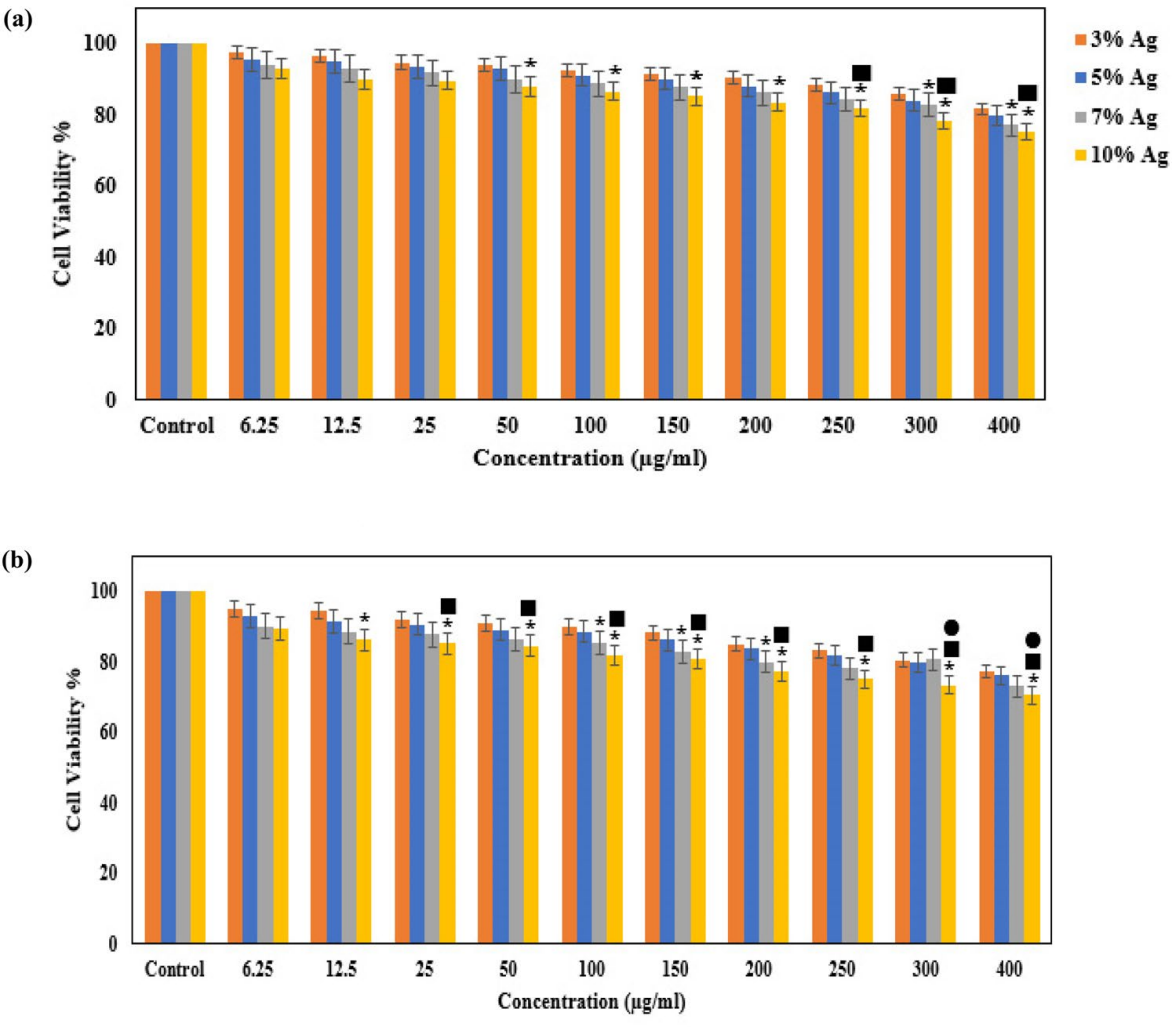
Evaluation of the cytotoxicity effect of different concentrations of Ru-Ag-MSN through the exposure time on fibroblast cells after (**a**) 24 and (**b**) 48 h.

The results of the MTT assay analysis indicated that Ag Ru-templated MSNs exhibit minimal cytotoxicity for normal fibroblast cells at the mentioned antibacterial and antifungal concentrations (Fig. 7). However, higher concentrations are still less toxic for normal fibroblast cells; that more than 80% and 75% of normal fibroblast cells were alive at the concentration of 250 µg/ml of 10% Ru-Ag-MSN after 24 and 48 h, respectively.

Silver nanoparticles can damage normal cells through mitochondrial or DNA destruction and increase reactive oxygen species that can result in cell death^65^.

Silica materials are demonstrated to have good biocompatibility, which makes them as a biocompatible platform for many biomedical applications^66–69^. Human normal cells usually have a neuter charge membrane with a low affinity for Ag NPs. Also, the slow release of Ag NPs from Ag Ru-templated MSNs makes it possible for normal human cells to activate defense mechanisms against ROS products of Ag NPs and escape from critical DNA damage.

We found that increasing the concentration of decorated Ag on the MSN platform to 10% could significantly improve the antimicrobial properties with low and acceptable cytotoxicity. This is closely depend on the unique structure of uniformly distributed small-sized Ag NPs (2 − 10 nm) on MSN platform that makes it possible to sustain the release of Ag NPs from the complex.

On the other hand, the addition of the flavonoid components of Ru extract to the Ag-MSN compound helps to produce homogeneous, distributed Ag nanoparticles on MSN background by acting as a reduction agent. That increased the antimicrobial properties with better cell safety and biocompatibility.

## Conclusions

It is a key path of contemporary nanotechnology investigation and implementation to develop Ag-decorated Ru-templated MSNs that are high-efficiency, low-cost, and environmentally friendly. According to the findings of this investigation, Ag NPs with homogenous distribution and small sizes were effectively decorated on Ru-templated MSNs for the first time. As compared to previous unmodified synthesis techniques, the present work offers a green synthesis approach for the fabrication of Ag-decorated MSNs that is cost-effective, nontoxic, and environmentally friendly. Furthermore, Ru-Ag-decorated MSNs revealed great antimicrobial efficacy against both gram negative and gram positive bacteria and diverse types of fungi with acceptable safety and low cytotoxicity. These findings suggest that Ag-decorated Ru-templated MSNs could be employed as excellent antimicrobial and biocompatible agents for various biomedical applications in the future. However, it requires supplementary in vivo and in vitro evaluations to confirm the efficacy or safety of Ru-Ag-MSN for medical applications.

## Supplementary Information

Supplementary Figures.

## Data Availability

All data generated or analysed during this study are included in this published article and its supplementary material files.
