# Supplementary material for: An intriguing approach toward antibacterial activity of green synthesized Rutin-templated mesoporous silica nanoparticles decorated with nanosilver
Source: Sci Rep. 2023 Apr 12;13:5987. doi: 10.1038/s41598-023-33095-1 (PMC10097644; doi:10.1038/s41598-023-33095-1)
Supplement: Supplementary file 1 — Supplementary Figures. [file 41598_2023_33095_MOESM1_ESM.docx]

**Supplementary Material**

**An intriguing approach toward antibacterial activity of green synthesized Rutin-templated mesoporous silica nanoparticles decorated with nanosilver**

Milad Abbasi^1†^, Razieh Gholizadeh^2†^, Seyed Reza Kasaee^3^, Ahmad Vaez^4^, Shreeshivadasan Chelliapan^5^, Fouad Fadhil Al-Qaim^6^, Issa Farhan Deyab^7^, Mostafa Shafiee^1^, Zahra Zareshahrabadi^8^, Ali Mohammad Amani^1*^, Sareh Mosleh-Shirazi ^2^*, Hesam Kamyab^9,10*^

^1^Department of Medical Nanotechnology, School of Advanced Medical Sciences and Technologies, Shiraz University of Medical Sciences, Shiraz, Iran

^2^ Department of Materials Science and Engineering, Shiraz University of Technology, Shiraz, Iran

^3^Shiraz Endocrinology and Metabolism Research Center, Shiraz University of Medical Sciences, Shiraz, Iran

^4^Department of Tissue Engineering and Applied Cell Sciences, School of Advanced Medical Sciences and Technologies, Shiraz University of Medical Sciences, Shiraz, Iran

^5^Engineering Department, Razak Faculty of Technology and Informatics, Universiti Teknologi Malaysia, Jln Sultan Yahya Petra, 54100, Kuala Lumpur, Malaysia

^6^College of Science for Women, University of Babylon, Hilla, Iraq

^7^Medical Physics Department, Al-Mustaqbal University College, 51001 Hillah, Babil, Iraq

^8^Basic Sciences in Infectious Diseases Research Center, Shiraz University of Medical Sciences,

Shiraz, Iran

^9^Malaysia-Japan International Institute of Technology (MJIIT), Universiti Teknologi Malaysia, Jalan Sultan Yahya Petra, 54100 Kuala Lumpur, Malaysia

^10^Department of Biomaterials, Saveetha Dental College and Hospital, Saveetha Institute of Medical and Technical Sciences, Chennai 600 077, India

*Corresponding Authors: Ali Mohammad Amani ([amani_a@sums.ac.ir](mailto:amani_a@sums.ac.ir)), Sareh Mosleh-Shirazi ([mosleh@sutech.ac.ir](mailto:mosleh@sutech.ac.ir)), Hesam Kamyab (hesam_kamyab@yahoo.com)

^†^ These authors contributed equally to the work.

**
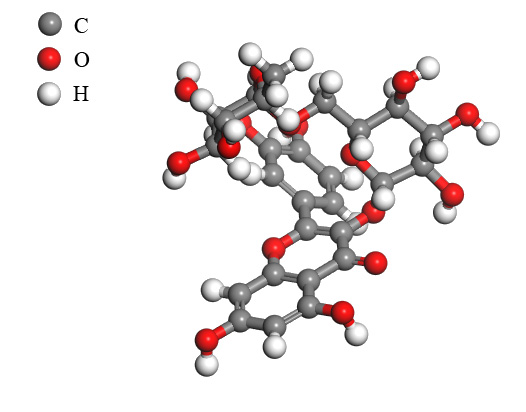
**

**Fig. S1** Molecular dynamic simulation of Rutin structure.


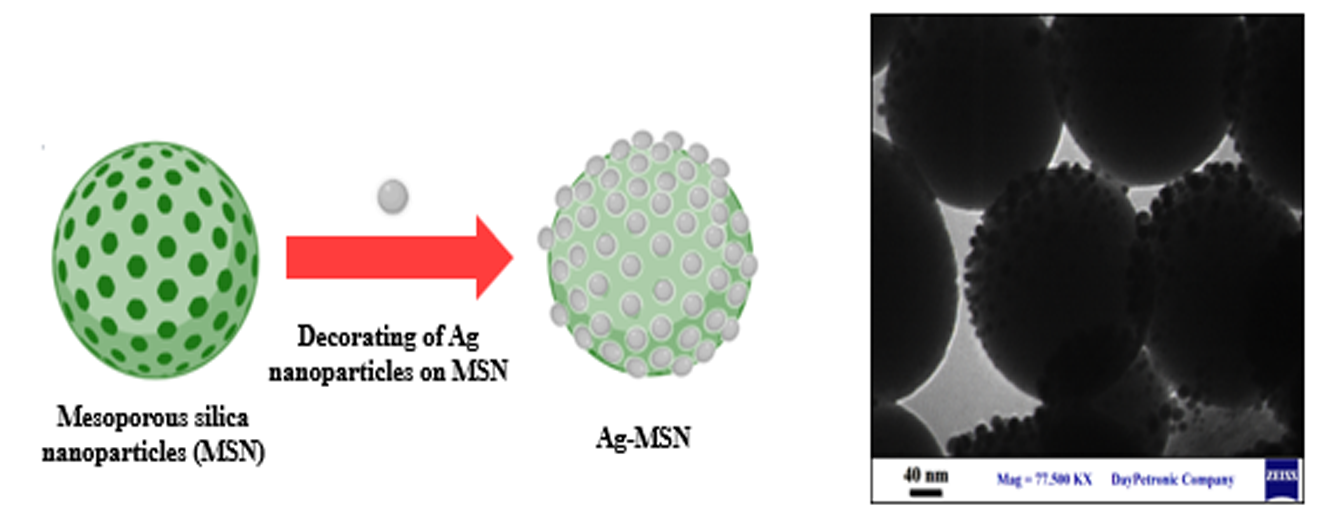


**Fig. S2** Schematic of decorating of Ag nanoparticles on MSN.

**
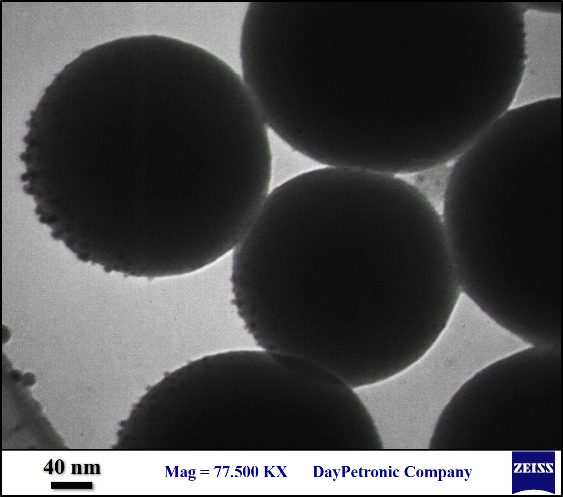

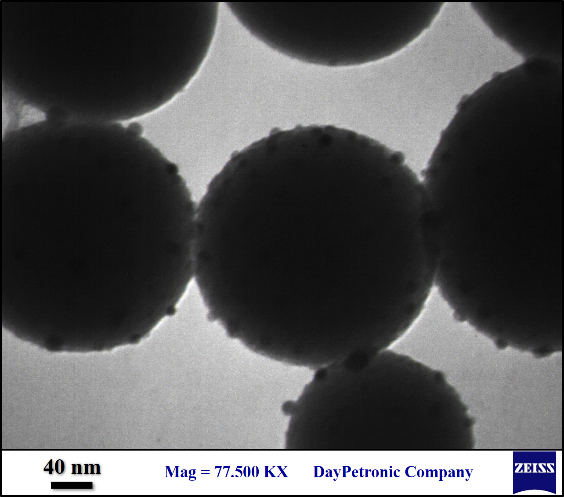
**

**(b)**

**(a)**

**
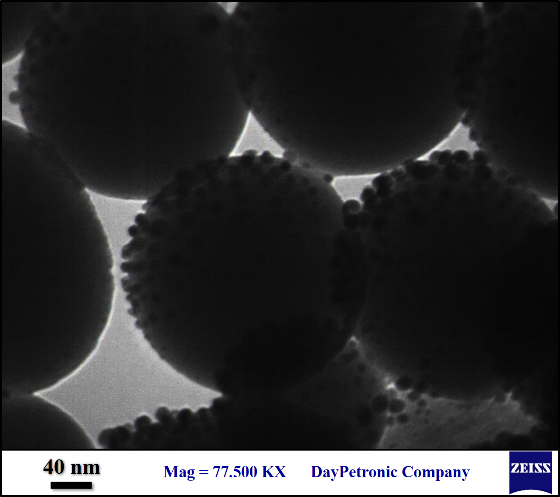

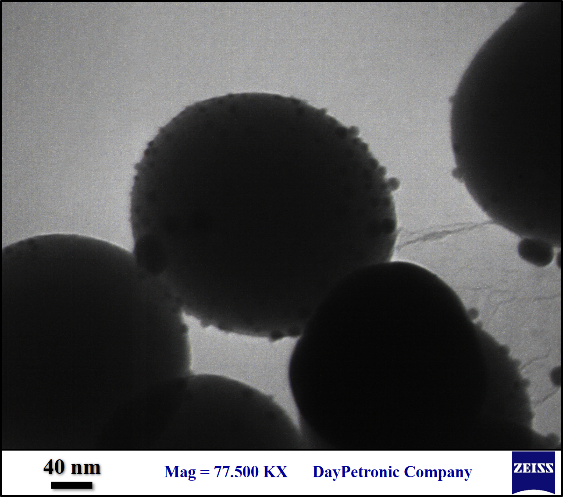
**

**(d)**

**(c)**

**Fig. S3** TEM micrographs of (a) 3, (b) 5, (c) 7, and (d) 10% Ag-decorated MSNs at 77500X magnification.


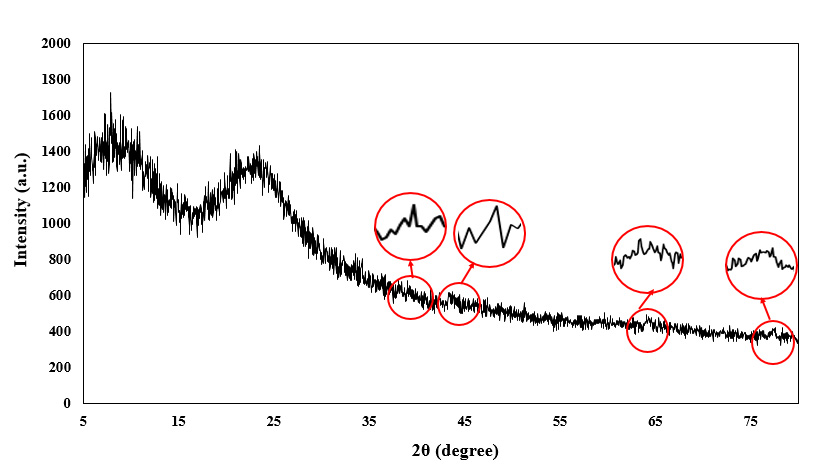


**Fig. S4** XRD pattern of 7% Ag-decorated MSNs.
